# Occurrence of *Escherichia coli* Pathotypes and Antimicrobial Resistance in Wastewater Effluent and Receiving Surface Waters in the Vhembe District, South Africa

**DOI:** 10.3390/microorganisms14051041

**Published:** 2026-05-04

**Authors:** Tshedza Mashamba, Johannes N. T. Mthembu, Vhukhudo Makhomu, Damien Jacobs, Mpumelelo Rikhotso, Leonard Kachienga, Natasha Potgieter, Afsatou N. Traore

**Affiliations:** Department of Biochemistry and Microbiology, Faculty of Science, Engineering and Agriculture, University of Venda, Thohoyandou 0950, South Africa; 21004987@mvula.univen.ac.za (T.M.); johannes.mthembu@univen.ac.za (J.N.T.M.); 20023995@mvula.univen.ac.za (V.M.); 17017009@mvula.univen.ac.za (D.J.); mpumelelo.rikhotso@univen.ac.za (M.R.); leonard.kachienga@univen.ac.za (L.K.); natasha.potgieter@univen.ac.za (N.P.)

**Keywords:** antimicrobial resistance, *E. coli*, mPCR, pathotypes, Vhembe district, WWTPs

## Abstract

Wastewater treatment plants (WWTPs) are identified as contributors to faecal pollution and the spread of antimicrobial resistance (AMR) in water ecosystems. This research examined the prevalence, profiles of antimicrobial resistance, and pathogenic types of *Escherichia coli* in effluent from WWTPs and nearby river systems in the Vhembe District. Between May and June 2025, 28 water samples were collected from two WWTP discharge points as well as upstream and downstream locations along the Mvudi, Luvuvhu, and Madadzhe Rivers. The enumeration of *E. coli* was conducted using Colilert Quanti-Tray method, with isolates obtained via membrane filtration and confirmed using API 20E and VITEK^®^2 systems. Antimicrobial susceptibility was assessed using VITEK^®^2, while pathotypes were detected through multiplex PCR. *E. coli* was found at all sampling locations; however, differences in concentrations across sampling sites and sampling periods were not statistically significant (*p* > 0.05). Out of 26 confirmed isolates, a significant resistance to β-lactam antibiotics was noted, especially ampicillin (100%). Pathotype analysis revealed strains such as ETEC, EAEC, and EPEC. These results underline extensive contamination by antimicrobial-resistant *E. coli* in rivers affected by WWTP discharge, which poses potential public health concerns and underscores the necessity for enhanced monitoring efforts. Additional research is needed to validate these findings.

## 1. Introduction

Surface water resources in Limpopo Province are critical for domestic, irrigation, and recreational use, especially in rural areas. These water sources are increasingly affected by inadequately treated effluent from wastewater treatment plants (WWTPs) due to operational inefficiencies, including hydraulic overloading, inadequate maintenance, and poor disinfection practices [1]. This raises significant public health concerns, as contaminated surface water may harbour pathogenic and antibiotic-resistant microorganisms [2].

Among microorganisms reported to be associated with contaminated water is *Escherichia coli* (*E. coli*). While many strains of *E. coli* are non-pathogenic, diarrheagenic pathotypes, such as enterotoxigenic *E. coli* (ETEC), enteroaggregative *E. coli* (EAEC), enteropathogenic *E. coli* (EPEC), and enterohaemorrhagic *E. coli* (EHEC), are associated with waterborne disease outbreaks [3]. The occurrence of these strains in the surface water is of concern as wastewater effluent can act as a reservoir and dissemination route [4]. Therefore, accurate detection and quantification of *E. coli* pathotypes in wastewater-impacted waters are essential for effective monitoring and risk assessment.

Studies conducted in Vhembe have documented faecal contamination and the presence of some diarrheagenic organisms in surface waters linked to wastewater treatment plants (WWTPs) [5]. However, these findings must be considered within the broader context of sanitation challenges in rural Vhembe District, where many households rely on on-site sanitation systems and, in some cases, untreated river water for domestic use, increasing the risk of faecal contamination [6,7]. These findings highlight the role of WWTPs as potential sources of faecal contamination when treatment processes are inadequate [8]. Of particular concern is the dissemination of antimicrobial-resistant bacteria, which can persist in the environment and contribute to the spread of waterborne diseases [9,10,11]. In addition to human health implications, these contaminants may alter aquatic microbial communities [12]. Therefore, effective operation and maintenance of wastewater treatment systems are essential to limit environmental and public health impacts.

Wastewater-influenced rivers are increasingly recognized as reservoirs of microbial contamination and antimicrobial-resistant bacteria, posing a significant public health risk to communities that rely on these waters for domestic use [13]. Data on the occurrence of diarrhoeagenic *E. coli* and their antimicrobial resistance profiles in the Vhembe district remain limited. However, studies across Africa and other low- and middle-income countries report a high prevalence of pathotypes such as EAEC and ETEC, often associated with increasing antimicrobial resistance, while wastewater treatment plants are widely recognized as important environmental reservoirs and hotspots for the dissemination of resistance genes [14,15]. Understanding the prevalence of *E. coli* pathotypes and resistance patterns in these surface waters is therefore critical to inform water quality management and public health interventions.

This study aimed to assess the occurrence, distribution, and antimicrobial resistance profiles of *E. coli* in wastewater-impacted surface waters within the Vhembe district. Specifically, the study compared two WWTPs with contrasting systems: an activated sludge system with chlorination and a pond-based system by assessing upstream, effluent, and downstream sites. A combination of Colilert enumeration, Vitek^®^ antibiotic susceptibility testing, and multiplex PCR was used to characterise isolates. In addition, the study assessed diverse pathotypes and resistance patterns, including those of clinical importance, to provide a comprehensive understanding of the influence of wastewater effluent on surface water microbial quality.

## 2. Materials and Methods

### 2.1. Study Design and Source of Samples

**Figure 1:**
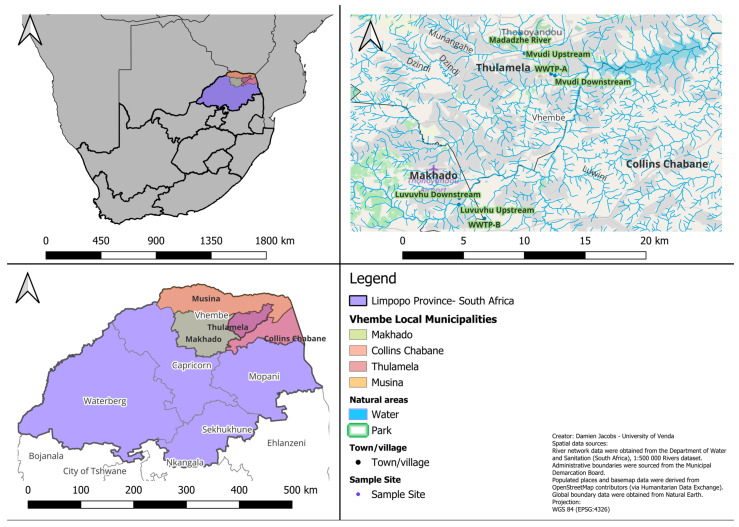
Map of the study in the Vhembe District, Limpopo Province, South Africa, showing the three systems (Mvudi, Luvuvhu, and Madadzhe) and associated sampling sites. Sampling points included upstream, WWTP effluent discharge point, and downstream locations influenced by treated effluent from WWTP-A and WWTP-B during May and June 2025.

A total of 28 water samples were collected from three rivers impacted by WWTP-A and WWTP-B in the Vhembe District of Limpopo Province, South Africa (Figure 1). Sampling was conducted across seven sites (upstream, effluent, and downstream) in duplicate per site over two sampling periods (May and June). Each water sample was prepared independently of presumptive *E. coli* using membrane filtration and selective culture. One representative colony per sample was selected for further identification, followed by biochemical and automated confirmatory identification. Although physicochemical parameters were recorded during sampling, these data are not included in the present study, which focuses on the microbiological assessment of *E. coli* and associated antimicrobial resistance profiles. WWTP-A uses a combination of activated sludge, drying beds, and trickling filters for water treatment. The water is disinfected through chlorination before discharge into the river. While WWTP-B uses a pond-based treatment system. Madadzhe River was included as a comparative site, although it is also impacted by direct sewage inputs.

### 2.2. Enumeration

Water samples were collected in sterile 500 mL polypropylene bottles (ALPLA, Hard, Austria) and transported to the laboratory on ice. All samples were processed within 6 h of collection to minimise changes in microbial composition. Enumeration of *E. coli* and total coliforms was performed using the Colilert Quanti-Tray/2000 kit (IDEXX Laboratories, Westbrook, ME, USA), following USEPA Method 9223B [16]. The mixture was placed in a sterile Quanti-Trays, sealed, and incubated at 37 °C for 24 h. After incubation, wells exhibiting a yellow colour were counted as positive for total coliforms, while wells showing fluorescence under UV light were recorded as positive for *E. coli*. Both large and small wells were counted separately, and the number of positive wells was used to determine the most probable number (MPN) per 100 mL (MPN/100 mL), using the manufacturer’s MPN table [17]. *E. coli* ATCC 25922 (Thermo Fisher Scientific, Waltham, MA, USA) and distilled water were used as positive and negative controls, respectively.

### 2.3. Isolation

Isolation of *E. coli* was performed by membrane filtration, as per standard methods for the examination of water [18,19]. Briefly, 100 mL of each water sample was filtered directly through a sterile 0.45 µm pore-size membrane filter (Merck KGaA, Darmstadt, Germany) using a vacuum filtration apparatus (Sigma-Aldrich, Burlington, MA, USA). The membranes were then aseptically placed on Eosin Methylene Blue (EMB) agar plates (Millipore Sigma, Burlington, VT, USA) and incubated aerobically at 37 °C for 24 h [20]. Representative colonies were subcultured onto nutrient agar plates to isolate pure cultures. Pure cultures were stored in 15% glycerol (Sigma-Aldrich, Darmstadt, Germany) at −80 °C to maintain viability for subsequent identification and characterization.

### 2.4. Identification

The presumptive *E. coli* isolates obtained by membrane filtration were subjected to Gram staining according to the standard protocol. Gram-negative bacilli were then identified biochemically using the API 20E kit (bioMérieux, Marcy-l’Étoile, France). Bacterial suspensions representing a 0.5 McFarland standard were prepared and inoculated into API 20E strips, which were incubated at 37 °C for 24 h. The inoculated strips were placed in the API incubation tray, and 5 mL of sterile distilled water was added to the tray to maintain a humid environment. Identification results were interpreted using APIweb™ version 5 (bioMérieux, Marcy-l’Étoile, France) and only identifications with ≥90% confidence were accepted [21]. Final confirmation of isolates was performed using the VITEK^®^ 2 Compact system (bioMérieux, Marcy-l’Étoile, France) and identifications with ≥90% probability were accepted. *E. coli* ATCC 25922 was included as a quality control strain to ensure accuracy of identification.

### 2.5. Characterisation of Pathotypes

Extraction of DNA from confirmed *E. coli* isolates obtained from Colilert-positive samples was performed using the Quick-DNA^TM^ miniprep kit (Zymo Research, Irvine, CA, USA). The Colilert-positive samples stored in sterile 5 mL microcentrifuge tubes (Sigma-Aldrich, St. Louis, MO, USA) were used directly for DNA extraction according to the manufacturer’s instructions [22]. Briefly, bacterial cells suspended overnight in Colilert reagent were heated to boiling, and the DNA was purified and eluted in 50 µL of nuclease-free water (Thermo Fisher Scientific, Waltham, MA, USA). The concentration and purity of the DNA were assessed using a NanoDrop™ 8000 version 2.3.1 (Thermo Fisher Scientific, Waltham, MA, USA), and samples with A260/280 ratios between 1.8 and 2.0 were considered suitable for downstream applications.

Molecular confirmation of *E. coli* isolates was performed by PCR amplification of the housekeeping malate dehydrogenase (*mdh*) gene [23]. Multiplex PCR assay was subsequently used to detect virulence genes of diarrheagenic *E. coli* pathotypes, such as *lt-1* and *st-α* (ETEC), *eae* (EPEC), *stx1* and *stx2* (EHEC), *ial* (EIEC), and EAEC (*eagg*) (Table 1) [23,24,25].

Polymerase chain reaction amplification was performed in a 25 µL reaction mixture containing 12.5 µL of 2 × PCR Master Mix (Thermo Fisher Scientific, Waltham, MA, USA), 0.2–0.4 µM of each gene-specific primer (Inqaba Biotech, Pretoria, South Africa), 2 µL of template DNA, and nuclease-free water (Thermo Fisher Scientific, Waltham, MA, USA). The thermal cycling parameters included an initial denaturation step of 95 °C for 5 min, followed by 30 cycles of denaturation at 95 °C for 30 s, annealing at 55–60 °C for 30 s, extension at 72 °C for 1 min, and a final extension of 10 min at 72 °C [26]. Positive controls for each target gene and a no-template negative control were included in all PCR runs to ensure assay validity. Amplified PCR products were resolved on a 1.5% (*w*/*v*) agarose gel prepared in 1× TBE buffer and stained with ethidium bromide (Sigma-Aldrich, St. Louis, MO, USA). The gel was illuminated under ultraviolet transillumination. A 100 bp DNA ladder (Thermo Fisher Scientific, Waltham, MA, USA) was used to estimate the amplicon sizes.

**Table 1:** Gene targets and primer sequences used for the identification of *E. coli* pathotypes.

Pathogen	Primer	Sequence (5′-3′)	Size (bp)	References
*E. coli*	*mdh* (F)	GGT ATG GAT CGT TCC GAC CT	300	[22]
	*mdh* (R)	GGC AGA ATG GTA ACA CCA GAG T	300	[22]
EIEC	*ial* (F)	GGT ATG ATG ATG ATG AGT CCA	650	[24]
	*ial* (R)	GGA GGC CAA CAA TTA TTT CC	650	[24]
EAEC	*eagg* (F)	AGA CTC TGG CGA AAG ACT GTA TC	194	[25]
	*eagg* (R)	ATG GCT GTC TGT AAT AGA TGA GAA C	194	[25]
EHEC	*stx 1* (F)	ACA CTG GAT GAT CTC AGT GG	614	[27]
	*stx 1* (R)	CTG AAT CCC CCT CCA TTA TG	614	[27]
	*stx 2* (F)	CCA TGA CAA CGG ACA GCA GTT	779	[27]
	*stx 2* (R)	CCT GTC AAC TGA GCA CTT TG	779	[27]
ETEC	*lt-1* (F)	TGG ATT CAT CAT GCA CCA CAA GG	360	[25]
	*lt-1* (R)	CCA TTT CTC TTT TGC CTG CCA TC	360	[25]
	*st-α* (F)	TTT CCC CTC TTT TAG TCA GTC AAC TG	160	[25]
	*st-α* (R)	GGC AGG ATT ACA ACA AAG TTC ACA	160	[25]
EPEC	*eae* (F)	CTG AAC GGC GAT TAC GCG AA	917	[23]
	*eae* (R)	GAC GAT ACG ATC CAG	917	[23]

### 2.6. Characterisation of Antimicrobial Resistance (AMR)

Antimicrobial susceptibility testing was performed on confirmed *E. coli* isolates using the VITEK^®^ 2 automated system (BioMérieux, Marcy-l’Étoile, France) with the AST Gram-negative 96 card, according to the manufacturer’s instructions [28,29]. Prior to testing, bacterial cultures were prepared in sterile saline and adjusted to a turbidity equivalent to 0.5 McFarland units (1.5 × 10^8^ CFU/mL) before inoculation. The AST-GN96 cards included a panel of antibiotics representing multiple classes, including β-lactams (Amoxicillin, ampicillin, piperacillin/tazobactam, cefuroxime/axetil, cefoxitin, cefotaxime, ceftazidime, cefepime, ertapenem, imipenem, meropenem), aminoglycosides (gentamicin, amikacin, tobramycin), fluoroquinolones (ciprofloxacin), tetracyclines (tigecycline), and folate pathway inhibitors (trimethoprim), and polymyxins (colistin). The VITEK^®^ 2 system automatically determined minimum inhibitory concentrations, and isolates were classified as susceptible, intermediate, or resistant according to the Clinical and Laboratory Standards Institute (CLSI) guidelines. The Multiple Antibiotic Resistance index for each isolate was calculated as the ratio of the number of antibiotics to which the isolate was resistant to the total number of antibiotics tested.

### 2.7. Data Analysis

All data collected were compiled in Microsoft Excel, and statistical analysis and graphical representation were performed using GraphPad Prism version 8.4.3 (686) (GraphPad Software, San Diego, CA, USA). The data were presented as mean ± standard deviation. Analysis of variance (ANOVA) was used to determine differences in *E. coli* counts across sites (upstream, downstream, and effluent/discharge point) and periods (May and June) for the Luvuvhu and Mvudi Rivers, as well as in wastewater effluent from the WWTP-A and WWTP-B. Descriptive analysis was used for data from the Madadzhe River, as sites were randomly selected. A *p*-value < 0.05 was considered statistically significant. Where a statistically significant effect was observed (*p* < 0.05), Tukey’s post hoc test was performed for pairwise comparisons between groups.

## 3. Results

### 3.1. Enumeration of Escherichia Coli Using the Colilert Method

**Figure 2:**
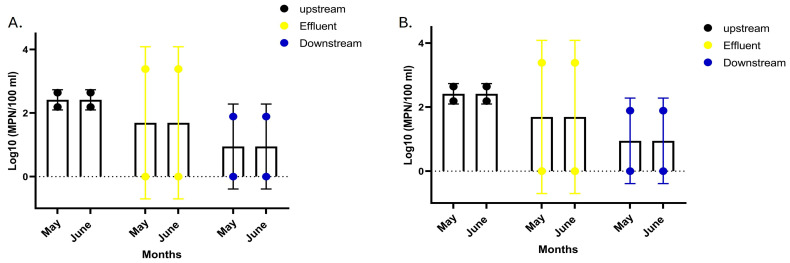
Log_10_ MPN/100 mL *E. coli* concentrations in (**A**) Mvudi River and (**B**) Luvuvhu River, presented as mean values (±standard deviation) for May and June across upstream, effluent discharge, and downstream sampling sites. Concentrations were determined using the Colilert Quanti-Tray system^®^. Overall, differences across rivers were not statistically significant (*p* > 0.05), although site-specific effects were observed in the Luvuvhu River (column factor, *p* = 0.0005).

In the Mvudi River, log_10_, MPN/100 mL *E. coli*, were found to be highest at effluent discharge sites for both May and June sampling periods (0–3.801) and lowest at downstream sites, recorded (0–2.101). Moderate concentrations (2.190–2.640) were found at upstream sites (Table A1). However, no significant differences were observed across the three sampling points in the Mvudi River (two-way ANOVA: column factor *p* = 0.472; interaction *p* > 0.999; Figure 2A).

In the Luvuvhu River, log_10_ MPN/100 mL *E. coli* MPN values, concentrations were highest at effluent discharge sites (3.383–3.581), whereas upstream showed concentrations of 1.571–2.250 (Table A1), and downstream sites showed lower concentrations (1.95–2.011) (Figure 2B). ANOVA revealed a significant effect for the column factor (*p* = 0.0005), indicating site-specific differences within this river. However, the row factor (*p* = 0.9998) and interaction (*p* > 0.9999) were not significant, and overall comparisons across rivers showed no statistically significant differences (*p* > 0.05).

**Figure 3:**
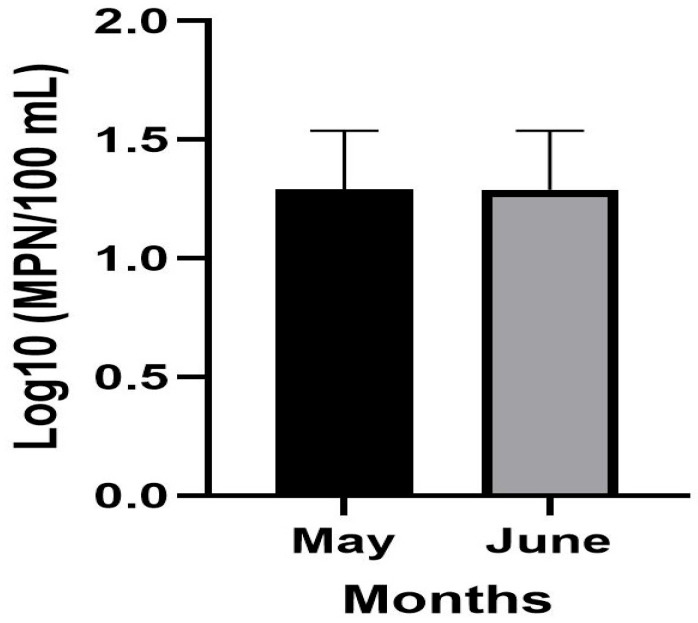
Log_10_ MPN/100 mL *E. coli* concentration in the Madadzhe River, presented as mean values (± standard deviation) for May and June, determined using the Colilert Quanti-Tray system^®^.

In the Madadzhe River, *E. coli* concentrations were relatively low and stable, with mean values of 1.290 and 1.291 log_10_ MPN/100 mL for May and June, respectively (Table A1). No significant variation was observed across sites (Figure 3). 

Overall, *E. coli* was detected at all sampling sites, with higher mean concentrations observed at effluent and downstream sites compared to upstream locations. However, these differences were not statistically significant (*p* > 0.05).

### 3.2. Isolation and Molecular Confirmation of E. coli

Presumptive *E. coli* colonies were obtained from surface water samples following membrane filtration and growth on selective media. A total of 28 isolates were obtained and subjected to biochemical identification using the API 20E system, followed by confirmatory identification using the VITEK^®^ 2 Compact system. Of the presumptive isolates, 26 (93%) were confirmed as *E. coli*, while a subset of isolates was identified as other members of the Enterobacteriaceae, including *Enterobacter cloacae* complex (Table A2). Although identified as *Enterobacter cloacae* complex rather than *E. coli*, these isolates were included in the AMR heatmaps to reflect the broader antimicrobial resistance profile of Enterobacteriaceae in the WWTP effluent. Confirmed *E. coli* isolates were recovered from all sampling sites across both sampling periods. However, not all duplicate samples yielded confirmed isolates or detectable pathotypes, resulting in some sites being represented by isolates with no detected pathotypes. Molecular confirmation targeting the *mdh* gene further verified the identity of the isolates selected for downstream analysis (Table A3 and Table 2). The 26 confirmed *E. coli* isolates were subsequently used for pathotype characterisation and antimicrobial susceptibility testing. Detailed identification results are provided in Appendix A Table A2.

### 3.3. Prevalence of E. coli Pathotypes

A total of 26 confirmed *E. coli* isolates were screened for virulence genes associated with five pathotypes. The most common pathotypes detected were ETEC and EAEC, each accounting for 42.31% (11/26) of isolates, followed by EPEC at 27% (7/26). EIEC and EHEC were less frequent, detected in 7.64% (2/26) and 3.84% (1/26) of isolates, respectively. Multiple pathotypes were observed in several isolates in the Luvuvhu River: upstream 2 contained ETEC, EPEC, EIEC (June); WWTP-B sites had EPEC and EAEC during May and June 2025; and Madadzhe 1 June carried a combination of all five pathotypes (ETEC, EAEC, EPEC, EHEC, EIEC). A notable proportion of the isolates exhibited hybrid characteristics, with 38.46% (10/26) carrying virulence genes associated with more than one diarrheagenic *E. coli* pathotype.

### 3.4. Antimicrobial Susceptibility of E. coli from Wastewater and Surface Water

**Figure 4:**
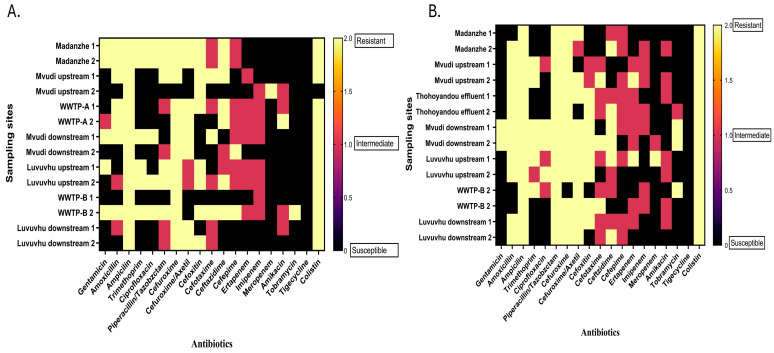
Heatmaps of antimicrobial susceptibility profiles of individual *E. coli* isolates from selected river sampling sites in the Vhembe district. (**A**) May 2025; (**B**) June 2025. Each cell represents the susceptibility result of a single isolate to a specific antibiotic. Colour coding indicates susceptibility patterns: black represents susceptible (S), pink intermediate (I), and yellow resistant (R) responses across the antibiotics tested.

The heat maps indicated widespread resistance patterns among *E. coli* isolates from all sites in both May and June. In May, higher resistance levels were observed for WWTP-A effluent, Mvudi downstream, Luvuvhu downstream, and WWTP-B effluent, whereas lower resistance levels were observed at upstream sites (Figure 4A). The resistance levels were predominantly observed with β-lactam antibiotics, including ampicillin (100%; 28/28), amoxicillin (64.3%; 18/28), and cefotaxime (21.43%; 6/28), and with folate pathway inhibitors, trimethoprim (43%; 12/28). Resistance to polymyxins, colistin (96%; 27/28), was observed in all isolates except Mvudi upstream 2, whereas most remained susceptible to tetracyclines (tigecycline) (100%; 28/28). Resistance to extended-spectrum cephalosporins was observed, as WWTP-B 2, Mvudi discharge and Mvudi upstream showed resistance to cefotaxime (21.43%; 6/28); Madadzhe, WWTP-A 1, Mvudi upstream 1 and 2, and WWTP-A 1 and 2 showed resistance to ceftazidime (46.4%; 13/28), and WWTP-B and Mvudi downstream 2 showed resistance to cefepime (7.14%; 2/28).

Overall, in both months, a high level of resistance across ESBLs and β-lactam and aminoglycoside antibiotic classes was observed, consistent with multidrug resistance profiles (Figure 4). Specific isolates, including those from WWTP-B (sample 2, May) (Figure 4A) and Mvudi downstream (sample 2, June) (Figure 4B), exhibited resistance to 13 antibiotics and were classified as extensively drug-resistant. In contrast, upstream isolates showed lower levels of resistance.

## 4. Discussion

This study aimed to assess *E. coli* occurrence and antimicrobial resistance in wastewater-impacted rivers, and the findings reveal widespread contamination across all sampling sites. The widespread detection of *E. coli* across all sampling sites in the Mvudi, Luvuvhu, and Madadzhe Rivers indicates pervasive faecal contamination within these systems. Although higher concentrations were observed downstream of WWTP discharge points, these differences were not statistically significant, suggesting that additional contamination sources or environmental factors may also contribute. Moreover, the widespread occurrence of *E. coli* suggests poor microbial water quality and faecal contamination, with concentrations exceeding recommended surface water limits, posing potential public health risks if the water is used untreated [30]. Similar trends have been reported both regionally and globally, highlighting the impact of wastewater effluent on surface water quality [31,32].

While river contamination exceeded surface water limits, treated wastewater effluent must comply with microbiological standards to protect receiving water bodies [33]. In this context, the elevated downstream concentrations, although not statistically significant, may suggest a contribution of WWTP effluent to faecal contamination, consistent with previous reports of increased microbial loads downstream of the discharge point [31,34,35]. This is particularly relevant in the Vhembe District, where communities frequently use river water for domestic activities, increasing the potential for human exposure.

To better understand the sources and extent of contamination, presumptive *E. coli* isolates from WWTP effluent and river samples were confirmed using selective culture and identification methods. Confirmatory identification revealed presumptive *E. coli* at all sampling sites; however, discrepancies of 7.69% (2/26) were observed, with misidentification occurring specifically in WWTP A effluent samples during June, where isolates were identified as *Enterobacter cloacae complex* rather than *E. coli* (Table A2). This suggests that the reliance on selective media alone can result in misclassification of closely related coliform bacteria, a phenomenon previously reported in studies showing that culture-based methods may overestimate *E. coli* prevalence due to phenotypic similarity among members of the Enterobacteriaceae family [36]. Notably, this was site-specific, occurring only in WWTP-A effluent, suggesting potential limitations during primary isolation or shifts in microbial community composition within the treatment system, favouring opportunistic *Enterobacter* spp. over *E. coli* [37,38]. Enterobacter species are opportunistic pathogens commonly found in water and soil and can cause urinary tract, respiratory tract, and bloodstream infections [39,40].

Confirmed *E. coli* isolates were analysed to assess the distribution of major diarrheagenic pathotypes. Five pathotypes were detected, with ETEC and EAEC (42.31%) most frequent, and EIEC and EHEC (7.69% and 3.84%, respectively) less common, indicating that the Vhembe rivers can serve as reservoirs of potentially infectious bacteria [41,42]. Moreover, studies have demonstrated that pathotype distributions varied regionally, with higher EPEC in Johannesburg [36] and ETEC dominance in Poland [42] and Tunisia [42] reflecting differences in contamination sources, water quality, and socio-environmental factors [43]. The detection of EAEC aligns with its persistence in water, while low EHEC and EIEC prevalence likely reflects limited circulation rather than poor environmental survival [43,44].

The predominance of ETEC and EAEC (both 42.3%) suggests that human faecal pollution, particularly in areas with poor sanitation and wastewater management [1]. Of particular concern is the detection of hybrid pathotypes, with 38.46% (10/26) of isolates harbouring virulence genes from multiple diarrheagenic *E. coli* groups. Such hybrid strains, including combinations of ETEC and EAEC virulence factors, have been reported in environmental and clinical settings and are associated with persistence and greater disease severity than single-pathotype strains [45,46]. Their presence in wastewater-impacted rivers suggests that WWTPs and receiving waters may act as mixing points for genetic exchange [11,47]. This could explain the predominance pattern of ETEC and EAEC observed in the current study.

In addition to *E. coli* pathogenic potential, these *E. coli* pathotypes exhibited high levels of antibiotic resistance. This was observed in environmental *E. coli* across all sampling sites and both months, with the highest resistance recorded against β-lactams [ampicillin 100% (28/28), amoxicillin 64.3 (18/28), cefotaxime 21.43% (6/28)], and trimethoprim 43% (12/28), as well as notable colistin resistance 96% (27/28). The presence of colistin-resistant strains is particularly concerning, as colistin is a last-resort antibiotic for multidrug-resistant Gram-negative infections [48,49]. Such high levels of resistance may be linked to plasmid-mediated colistin resistance determinants, including members of the *mcr* gene family, which have been increasingly reported in environmental and wastewater-associated *E. coli* [50,51]. However, these mechanisms were not investigated in the present study, and further molecular characterization would be necessary to confirm their presence and better understand their potential for environmental dissemination. These resistance patterns likely suggest selective pressure from environmental antimicrobial compounds, including inputs from WWTP effluents and other anthropogenic sources. Indeed, studies in South Africa have detected a wide range of antibiotic residues in wastewater effluents and receiving rivers, often at concentrations ranging from ng/L to µg/L, which can contribute to the selection of resistant bacteria [52]. In addition, co-selection mechanisms involving heavy metals and disinfectants commonly used in wastewater treatment processes may further promote the persistence and dissemination of antimicrobial resistance in aquatic environments [53]. Although this study did not assess underlying mechanisms such as ESBL production, the high levels of resistance to β-lactam antibiotics may be indicative of extended-spectrum β-lactamase (ESBL)-associated genes such as *bla_TEM_* and *bla_CTX-M_*, which are commonly reported in wastewater and environmental *E. coli* isolates [54]. In addition, plasmid-mediated mechanisms are known to facilitate the dissemination of these resistance determinants in aquatic environments, particularly in wastewater-impacted systems [55,56]. However, the absence of genotypic analysis in the present study limits the ability to confirm these mechanisms, and further molecular investigations are required. Consistent with this, similar studies have reported widespread resistance genes, including *bla_TEM_* and *tetA/B*, among multidrug-resistant *E. coli* in South African surface waters and effluent-impacted sites [1,50,57]. Comparable trends have been observed in other studies with higher abundances of resistant *E. coli* downstream of WWTPs reported in rivers such as the Svratka River in the Czech Republic, likely due to environmental dispersion and hydrological factors [58,59,60,61,62].

This study had some limitations, such as a small number of confirmed *E. coli* isolates (*n* = 26), which may limit the robustness and generalisability of conclusions regarding pathotype distribution and antimicrobial resistance patterns. Sampling was conducted over a short period (May–June 2025) and therefore does not capture potential seasonal variation. In addition, although resistance to multiple antibiotics was observed, the underlying mechanisms, including ESBL production and resistance genes, were not investigated. Furthermore, the use of culture-based methods and phenotypic antimicrobial susceptibility testing alone does not allow differentiation between intrinsic, mutational, or horizontally acquired resistance mechanisms. Future studies should incorporate molecular approaches, such as whole genome sequencing of selected multidrug-resistant isolates, to better characterize resistance determinants and their potential for environmental dissemination. Furthermore, the Madadzhe River, included as a comparative site, is impacted by sewage inputs and does not represent a true unimpacted control. Future studies should incorporate longitudinal sampling, molecular characterisation, and appropriate control sites.

## 5. Conclusions

This study demonstrates the ubiquity of *E. coli* in the surface waters of the Vhembe District, with concentrations exceeding recommended limits, indicating significant faecal contamination from wastewater discharge and other anthropogenic activities. The detection of diarrhoeagenic *E. coli* pathotypes, such as ETEC and EAEC, indicates potential health risks posed by community-wide use of these surface waters. The high rates of antimicrobial resistance, including antibiotic resistance, indicate the dissemination of clinically relevant resistance mechanisms in the environment and the presence of mobile genetic elements that facilitate their spread. The detection of resistant *E. coli* in both effluent and surface waters supports the notion that WWTPs may act as potential reservoirs and dissemination pathways for antibiotic-resistant bacteria, potentially facilitated by horizontal gene transfer mechanisms such as plasmids and integrons. Overall, these findings emphasize the need for improved wastewater treatment practices and continuous monitoring to mitigate the spread of antimicrobial resistance in aquatic environments.

## Figures and Tables

**Table 2:** Occurrence of *E. coli* pathotypes detected by mPCR across sampling sites and months in surface waters of the Vhembe District, South Africa.

Sample ID	Month	*E. coli* Pathotype
Mvudi upstream 1	May	EPEC
Mvudi upstream 2	May	ETEC, EAEC
Mvudi upstream 1	June	No pathotype detected
Mvudi upstream 2	June	ETEC, EAEC
WWTP-A 1	June	No pathotype detected
WWTP-A 2	June	No pathotype detected
Mvudi downstream 1	May	No pathotype detected
Mvudi downstream 2	May	No pathotype detected
Mvudi downstream 1	June	No pathotype detected
Mvudi downstream 2	June	No pathotype detected
Luvuvhu upstream 1	May	EAEC
Luvuvhu upstream 2	May	EAEC, ETEC
Luvuvhu upstream 1	June	ETEC, EAEC
Luvuvhu upstream 2	June	ETEC, EPEC, EIEC
WWTP-B 1	May	EPEC, EAEC
WWTP-B 2	May	ETEC
WWTP-B 1	June	EAEC
WWTP-B 2	June	EPEC, EAEC
Luvuvhu downstream	May	ETEC
Luvuvhu downstream	May	EPEC, ETEC, EAEC
Luvuvhu downstream	June	EPEC, ETEC, EAEC
Luvuvhu downstream	June	ETEC
Madadzhe 1	May	No pathotype detected
Madadzhe 2	May	No pathotype detected
Madadzhe 1	June	EPEC, EHEC, ETEC, EIEC, EAEC
Madadzhe 2	June	No pathotype detected

## Data Availability

The original contributions presented in this study, including raw MPN values, mPCR characterization, and AST data, are available from the corresponding author at reasonable request.

## Abbreviations

The following abbreviations are used in this manuscript:

API	Analytical Profiling Index
*E. coli*	*Escherichia coli*
EMB	Eosin Methylene Blue
EAEC	Enteroaggregative *Escherichia coli*
EHEC	Enterohemorrhagic *Escherichia coli*
EIEC	Enteroinvasive *Escherichia coli*
ETEC	Enterotoxigenic *Escherichia coli*
EPEC	Enteropathogenic *Escherichia coli*
ESBLs	Extended Spectrum β Lactams
MPN	Most Probable Number
WWTP	Wastewater treatment plant

## Appendix A

**Table A1:** The Log_10 MPN_/_100 mL_ *E. coli* (May and June 2025).

Sampling Sites	May	June
Mvudi upstream 1	2.64	2.19
Mvudi upstream 2	2.19	2.64
WWTP-A 1	3.38	3.38
WWTP-A 2	0.00	0.00
Mvudi downstream 1	0.00	0.00
Mvudi downstream 2	2.00	2.00
Luvuvhu upstream 1	1.58	2.25
Luvuvhu upstream 2	1.58	2.25
WWTP-B 1	3.38	3.38
WWTP-B 2	3.38	3.38
Luvuvhu downstream 1	2.02	2.02
Luvuvhu downstream 2	1.96	1.96

**Table A2:** Bacterial species confirmed by VITEK^®^2 in environmental water samples from the Vhembe District across sampling months.

Sample ID	Month	Species
Madadzhe 1	May	*E. coli*
Madadzhe 1	June	*E. coli*
Madadzhe 2	May	*E. coli*
Madadddzhe 2	June	*E. coli*
Mvudi upstream 1	May	*E. coli*
Mvudi upstream 1	June	*E. coli*
Mvudi upstream 2	May	*E. coli*
Mvudi upstream 2	June	*E. coli*
WWTP-A effluent 1	May	*E. coli*
WWTP-A effluent 1	June	*Enterobacter cloacae complex*
WWTP-A effluent 2	May	*E. coli*
WWTP-A effluent 2	June	*Enterobacter cloacae complex*
Mvudi downstream 1	May	*E. coli*
Mvudi downstream 1	June	*E. coli*
Mvudi downstream 2	May	*E. coli*
Mvudi downstream 2	June	*E. coli*
Luvuvhu Upstream 1	May	*E. coli*
Luvuvhu Upstream 1	June	*E. coli*
Luvuvhu Upstream 2	May	*E. coli*
Luvuvhu Upstream 2	June	*E. coli*
WWTP-B effluent 1	May	*E. coli*
WWTP-B effluent 1	June	*E. coli*
WWTP-B effluent 2	May	*E. coli*
WWTP-B effluent 2	June	*E. coli*
Luvuvhu downstream 1	May	*E. coli*
Luvuvhu downstream 1	June	*E. coli*
Luvuvhu downstream 2	May	*E. coli*
Luvuvhu downstream 2	June	*E. coli*

**Table A3:** Virulence gene profiles of environmental diarrheagenic *E. coli* isolates detected by multiplex PCR.

SAMPLE NAME				EHEC							
			EPEC				EIEC	ETEC		EAEC	Virulence Genes Detected
	*Asta*	*mdh*	*Bfp*	*Eae*	*stx1*	*stx2*	*Ial*	*lt-1*	*st-α*	*eagg*	
Levubu downstream 1 (05)	-	+	-	-	-	-	-	+	-	-	*lt-1*
Levubu downstream 2 (05)	-	+	-	+	-	-	-	+	-	+	*Eae*, *lt-1*, *eagg*
Levubu effluent 2 (05)	-	-	-	-	-	-	-	+	-	-	*lt-1*
Levubu upstream 1 (05)	+	+	-	-	-	-	-	-	-	-	*asta*
Levubu upstream 2 (05)	+	+	-	-	-	-	-	+	-	+	*lt-1*, *eagg*
Mvudi downstream 2 (05)	-	-	-	-	-	-	-	-	-	-	
Levubu effluent 1 (05)	+	+	+	-	-	-	-	-	-	+	*asta*, *Bfp*, *eagg*
Madadzhe 1 (05)	-	+	-	-	-	-	-	-	-	-	
Madadzhe 2 (05)	-	+	-	-	-	-	-	-	-	-	
Mvudi downstream 1 (05)	-	+	-	-	-	-	-	-	-	-	
Mvudi upstream 1 (05)		+	-	+	-	-	-	-	-		*Eae*
Mvudi upstream 2 (05)	+	+	-	-	-	-	-	+	-	+	*lt-1*, *eagg*
	-	-	-	-	-	-	-	-	-	-	
Levubu downstream1 (06)	+	+	+	-	-	-	-	-	+	-	*asta*, *Bfp*, *st-α*
Levubu downstream 2		+	-	-	-	-	-	+	+	-	*st-α*
Levubu effluent 1 (06)	+	+	-	-	-	-	-	-	-	-	*asta*
Levubu effluent 2 (06)	+	+	-	+	-	-	-	-	-	-	*asta*, *Eae*
Levubu upstream1 (06)	+	+	-	-	-	-	-	-	+	+	*asta*, *st-α*, *eagg*
Levubu upstream 2 (06)	-	+	+	+	-	-	+	-	+	-	*Bfp*, *Eae*, *ial*, *st-α*
Madadzhe 1 (06)	-	+	+	+	-	+	-	+	+	+	*Bfp*, *Eae*, *stx2*, *lt-1*, *st-α*, *eagg*
Madadzhe 2 (06)	+	-	-	-	-	-	-	-	-	-	*asta*
Mvudi downstream1 (06)	-	-	-	-	-	-	-	-	-	-	
Thohoyandou effluent 1 (06)	-	-	-	-	-	-	-	-	-	-	
Thohoyandou effluent 2 (06)	-	-	-	-	-	-	-	-	-	-	
Mvudi upstream 1 (06)	-	+	-	-	-	-	-	-	-	-	

(+): Gene detected; (-): Not detected.
